# Infecting human hematopoietic stem and progenitor cells with SARS-CoV-2

**DOI:** 10.1016/j.xpro.2021.100903

**Published:** 2021-10-06

**Authors:** Hector Huerga Encabo, Rachel Ulferts, Aneesh Sharma, Rupert Beale, Dominique Bonnet

**Affiliations:** 1 Hematopoietic Stem Cell Laboratory, The Francis Crick Institute, London, NW1 1AT, UK; 2 Cell Biology of Infection Laboratory, The Francis Crick Institute, London NW1 1AT, UK

**Keywords:** Cell Biology, Cell isolation, Cell-based Assays, Immunology, Microbiology, Microscopy, Stem Cells

## Abstract

Determining how hematopoietic stem and progenitor cells (HSPCs) can be infected by viruses is necessary to understand and predict how the immune system will drive the host response. We present here a protocol to analyze the capacity of SARS-CoV-2 to infect different subsets of human HSPCs, inlcuding procedures for SARS-CoV-2 production and titration, isolation of human HSPCs from different sources (bone marrow, umbilical cord, or peripheral blood), and quantification of SARS-Cov-2 infection capacity by RT-qPCR and colony forming unit assay.

For complete details on the use and execution of this protocol, please refer to Huerga Encabo et al. (2021)

## Highlights

• Production and titration of SARS-CoV-2
• Isolation of human hematopoietic stem and progenitor cells from different sources
• Analysis of the infection capacity of SARS-CoV-2 in HSPCs
• Despite their intrinsic antiviral protection HSPCs can be infected by SARS-CoV-2

## Graphical abstract

**Figure:**
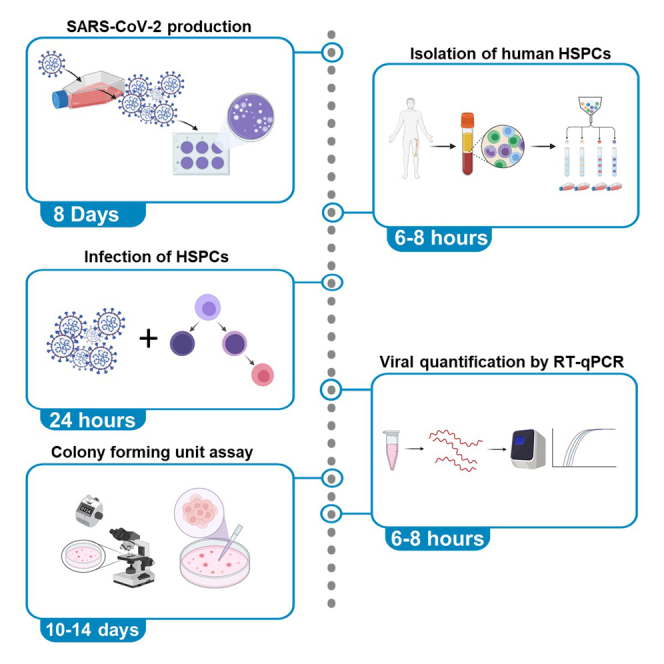

## Teaser

Determining how hematopoietic stem and progenitor cells (HSPCs) can be infected by viruses is necessary to understand and predict how the immune system will drive the host response. We present here a protocol to analyze the capacity of SARS-CoV-2 to infect different subsets of human HSPCs, inlcuding procedures for SARS-CoV-2 production and titration, isolation of human HSPCs from different sources (bone marrow, umbilical cord or peripheral blood), and quantification of SARS-Cov-2 infection capacity by RT-PCR and colony forming unit assay.

## Before you begin

**CRITICAL:** SARS-CoV-2 and any material that has been in contact with the virus should be handled and disposed of in accordance with your local regulations.

**CRITICAL:** Institutional permission to manipulate human samples should be approved before starting the protocol.

Ensure you have availability of a suitable source of HSPCs (i.e., cord blood, bone marrow or peripheral blood). In the case of processing fresh samples you should be processing a sample that has been acquired within the last 24 h. In the case of processing cryopreserved mononuclear cell suspension you should proceed from step 53 for the isolation of hematopoietic stem and progenitor cells (HSPCs) populations.

The isolation of primary human stem and progenitor hematopoietic cells under sterile conditions has been optimized in this protocol using FACSAria III in a CL2 cabinet.A

## Key resources table

**Table:** 

REAGENT or RESOURCE	SOURCE	IDENTIFIER
**Antibodies**		

CD34-PECy7 (dilution factor for staining 1/100)	eBioscience	clone 4H11
CD34- PerCP-Cy5.5 (dilution factor for staining 1/50)	BD Pharmingen	clone 8G12
CD38-PE (dilution factor for staining 1/50)	BD Pharmingen	clone HIT2
CD117-BB700 (dilution factor for staining 1/100)	BD Bioscience	clone YB5.B8
CD117-PECy7 (dilution factor for staining 1/100)	eBioscience	clone 104D2
CD71-APC (dilution factor for staining 1/100)	eBioscience	clone OKT9
CD235a-FITC (dilution factor for staining 1/100)	BD Pharmingen	clone HIR2
CD14-APCCy7 (part of Lineage exclusion gate) (1/100)	BioLegend	clone M5E2
CD16-APCCy7 (part of Lineage exclusion gate) (1/100)	BioLegend	clone B73.1
CD19-APCCy7 (part of Lineage exclusion gate) (1/100)	BioLegend	clone HIB19
CD3-APCCy7 (part of Lineage exclusion gate) (1/100)	BioLegend	clone SK7

**Bacterial and virus strains**		

SARS-CoV-2 isolate	(Huerga Encabo et al., 2021)(Huerga Encabo et al., 2021)	NA

**Biological samples**		

Human umbilical cord blood	Royal London Hospital (London, U.K.)	NA
Human peripheral blood from males and females between 20 and 50 years old	Francis Crick Institute	NA
Human bone marrow aspirates from males and females between 30 and 40 years old	STEMCELL Technologies	Cat#70001

**Chemicals, peptides, and recombinant proteins**		

Ficoll	GE Healthcare/Cytiva	Cat#17-1440-03
Ammonium Chloride (Red Cell Lysis Buffer)	Francis Crick Institute	NA
Fetal Bovine Serum	Gibco	Cat#10270-106
Dimethyl Sulfoxide (DMSO)	Sigma-Aldrich	Cat#D4540
MethoCult H4435 (100 mL)	STEMCELL Technologies	Cat#04435
Stemspan SFEM II	STEMCELL Technologies	Cat#9605
Human TPO	PeproTech	Cat#300-18
Human SCF	PeproTech	Cat#300-07
Human Flt3-Ligand	PeproTech	Cat#300-19
Avicell, Pharma grade RC581	IMCD	N/A
DMEM, high glucose, with glutamine	Thermo Fisher Scientific	Cat#11995065
FCS	Sigma	Cat#F7524
Formaldehyde solution for molecular biology, 36.5%–38% in H_2_O	Sigma	Cat#F8775
Triton-X100	Sigma	Cat#T9284
NaCl	Francis Crick Institute	NA
KCl	Francis Crick Institute	NA
Na_2_HPO_4_	Francis Crick Institute	NA
KH_2_PO_4_	Francis Crick Institute	NA
Na2 EDTA	Francis Crick Institute	NA
HEPES, 1M Buffer Solution	Thermo Fisher Scientific	Cat#15630049
Trypsin-EDTA (0.5%)	Thermo Fisher Scientific	Cat#15400054
100**×** Pen/strep (optional)	Thermo Fisher Scientific	Cat#15140122
Toluidine Blue O	Sigma	Cat#T3260
DAPI	BD Biosciences	Cat#564907
PowerUP SYBR Green	Applied Biosystems	Cat#15310939

**Critical commercial assays**		

EasySep Human Progenitor Cell Enrichment Kit II	STEMCELL Technologies	Cat#17936
RNeasy Microkit	QIAGEN	Cat#74004
SuperScript III First-Strand Synthesis	Thermo Fisher Scientific	Cat#18080051

**Experimental models: Cell lines**		

Vero E6 cells [Vero C1008, Vero 76, clone E6, Vero E6]	ECACC ATCC	85020206 CRL-1586

**Oligonucleotides**		

ACE2_forward:5′-GCTGCACAACCTTTTCTGCT-3′	Invitrogen	NA
ACE2_reverse:5′-AAATGCTTAGGTGTGGCTGC-3′	Invitrogen	NA
TMPRSS2_forward: 5′-GTACCTGCATCAACCCCTCT-3′	Invitrogen	NA
TMPRSS2_reverse: 5′-TATAGCCCATGTCCCTGCAG-3′	Invitrogen	NA
SARS-CoV2_N_forward: 5′-CACATTGGCACCCGCAATC-3′	Invitrogen	NA
SARS-CoV2_N_reverse: 5′-GAGGAACGAGAAGAGGCTTG-3′	Invitrogen	NA
SARS-CoV2_E_forward: 5′-ACAGGTACGTTAATAGTTAATAGCGT-3′	Invitrogen	NA
SARS-CoV2_E_reverse: 5′-ATATTGCAGCAGTACGCACACA-3′	Invitrogen	NA
SARS-CoV2_RdP_forward: 5′-GTGARATGGTCATGTGTGGCGG-3′	Invitrogen	NA
SARS-CoV2_RdP_reverse: 5′-CARATGTTAAASACACTATTAGCATA-3′	Invitrogen	NA
GAPDH_forward: 5′-GGAGCGAGATCCCTCCAAAAT-3′	Invitrogen	NA
GAPDH_reverse: 5′-GGCTGTTGTCATACTTCTCATGG-3′	Invitrogen	NA

**Software and algorithms**		

QuantStudio Real-Time PCR Software v1.7.1	Applied Biosystems	NA

**Other**		

Falcon® 50 mL Tubes	Corning	Cat#352070
T175 Flask	Thermo Fisher Scientific	Cat#159910
2 mL Syringe	BD (Emerald)	Cat#307727
Sterican MIX Blunt Fill Needle	B. Braun	Cat#4038088-01
TC-Treated 6, 12 or 48 well plate	Corning	Cat#CLS3516- Cat#CLS3513- Cat#CLS3548
Falcon® 5 mL Polypropylene Test Tubes	Corning	Cat#352063
0.2 mL PCR Tubes	Bio-Rad	Cat#TFI0201
Eppendorf Safe-Lock Tubes, 1.5 mL	Eppendorf	Cat#0030120086
EasySep Magnet	STEMCELL Technologies	Cat#18000
MicroAmp Optical 384-Well Reaction Plate	Applied Biosystems	Cat#4309849
FACSAria III	Becton Dickinson	NA

## Materials and equipment

**Table:** Fixative solution/ 10% neutral buffered formalin

Reagent	Final concentration	Amount
Formaldehyde solution for molecular biology, 36.5–38% in H_2_O	∼3.6%	10 mL
PBS	n/a	90 mL
**Total**	**n/a**	**100 mL**

Make fresh on day of use and keep at room temperature (RT; 20°C–25°C)

**Table:** Complete growth medium for Vero E6 cells/ D10

Reagent	Final concentration	Amount
DMEM	n/a	450 mL
FCS	10%	50 mL
**Total**	**n/a**	**500 mL**

Store at 4°C, use within 4 weeks, bring to 37°C prior to use

**Table:** Virus growth medium / D2

Reagent	Final concentration	Amount
DMEM	n/a	48 mL
FCS	2%	1 mL
1 M Hepes	20 mM	1 mL
**Total**	**n/a**	**50 mL**

Store at 4°C, use within 4 weeks, bring to 37°C prior to use

**Table:** Plaque assay overlay, volume per 6-well plate

Reagent	Final concentration	Amount
Virus growth medium	0.5**×**	6.5 mL
2.4% Avicel in water	1.2%	6.5 mL
**Total**	**n/a**	**13 mL**

Store at 4°C, use within 4 weeks, bring to 37°C prior to use

**Table:** 2.4% avicel in water

Reagent	Final concentration	Amount
Avicel	2.4**×**	1.2 g
Water (MQ)	n/a	500 mL
**Total**	**n/a**	**500 mL**

Stir the water vigorously while slowly adding the avicel to avoid clumps from forming, sterilize by autoclaving on a liquid cycle, store at room temperature (RT; 20°C–25°C), use within 3 month, redistribute by swirling vigorously prior to use

**Table:** 1**×** PBS

Reagent	Final concentration	Amount
NaCl		8 g
KCl		0.25 g
Na_2_HPO_4_		1.437 g
KH_2_PO_4_		0.25 g
Dist. Water (make up to)	n/a	Up to 1000 mL
**Total**	**n/a**	**1000 mL**

Sterilize by autoclaving on a liquid cycle, store at RT, use within 1 year

**Table:** 1**×** staining solution

Reagent	Final concentration	Amount
Toluidine blue	0.1% (w/v)	0.5 g
Triton X-100	0.1% (v/v)	0.5 mL
Dist. Water (make up to)	n/a	Up to 500 mL
**Total**	**n/a**	**500 mL**

Store at RT for up to 1 year

**Table:** Red Cell Lysis Buffer

Reagent	Final concentration	Amount
NH_4_Cl	0.15M	80.24 g
KHCO_3_	10 mM	10.012 g
Na_2_ EDTA	0.1 mM	0.372 g
Dist. Water (make up to)	n/a	Up to 10000 mL
**Total**	**n/a**	10000 mL

Store at 4°C for up to 1 year

**Table:** PBS+2%FBS

Reagent	Final concentration	Amount
FBS	2%	10 mL
1**×** PBS (make up to)	n/a	Up to 500 mL
**Total**	n/a	500 mL

Store at 4°C and use within 4 weeks

## Step-by-step method details

### Preparation of virus stock

**Timing: [4 days]**

This step enables the propagation of a defined reference virus strain in a standard cell line, Vero E6. Infectivity of the resultant virus stock will then be determined in the subsequent section (beginning at step 19).

1. For each 15 mL of virus stock to be produced prepare one 90% confluent 75 cm^2^ flask of Vero E6 cells. Prepare one additional flask per experiment for the mock control.

***Note:*** Properties of cell lines can change during prolonged passaging. Prepare stocks of early passages of your cell line and store in liquid nitrogen. Regularly (∼20 passages, or if changes to morphology of the cell line or infectability are observed) restart your cell culture with an early passage vial.

**Figure 1:**
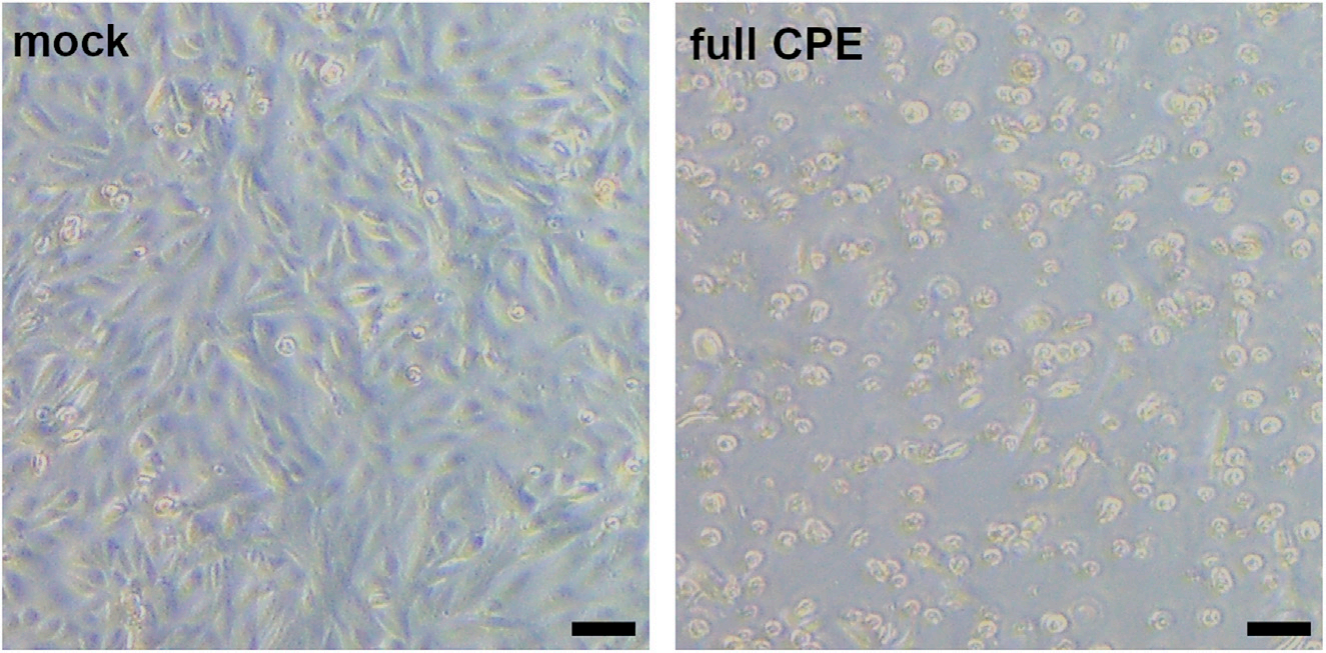
Phase contrast images showing a representative example of cytophatic effect (full CPE) in Vero E6 cells compared to control (mock) Scale bar 50 μm.

2. Calculate the required volume of virus stock (inoculum) for a multiplicity of infection (MOI) of 0.01 PFU per cell.
  a. A 75 cm^2^ flask of Vero E6 cells will contain approximately 10^7^ cells.
  b. The aim is to inoculate at a low multiplicity of infection (MOI, i.e., <0.01 PFU per cell) to avoid the build-up of defective-interfering particles during sequential subculture. A lower MOI than 0.01 can be used as determined by the user of this protocol.
3. Calculate the required volume of D2 medium (3 mL minus inoculum per 75 cm^2^ flask) and add this to a 5 mL reagent tube.
4. Add the inoculum to the reagent tube from step 3.
5. Remove the supernatant from the flasks.
6. Add the diluted inoculum to all flasks to be inoculated.
7. Add 3 mL of D3 medium to the mock control flask.
8. Incubate 30 min inside the microbiolocial safety cabinet to allow the virus to attach. Redistribute the supernatant repeatedly to prevent the cells from drying out.
9. Remove the supernatant and dispose as infectious waste according to local regulations.
10. Rinse all flasks twice with 10 mL D2.
11. Add 15 mL D2 to each flask.
  a. Incubate at 37°C and 5% CO_2_.
12. Starting from 2 days post infection (p.i.) observe the cell monolayers for signs of cytopathic effect (CPE) twice a day (morning and afternoon) using the inverted phase contrast microscope. The mock control flask serves as a comparison for an uninfected cell layer (see Figure 1).

**CRITICAL:** No CPE should be visible in the mock control flask. CPE in the control flask indicates poor sterile technique. This is particularly important if different strains or isolates are grown at the same time.

**CRITICAL:** Harvesting before obvious cytopathic effects risks a very low titer. Harvesting long after peak production risks losing infectivity due to thermal instability of the virus.

13. Once fulminant CPE is observed, transfer the supernatant into a centrifuge tube.
  a. Dispose of the flasks and remaining cells in accordance with local safety regulations.
14. Transfer the supernatant of the mock control flask into another centrifuge tube.
  a. Dispose of the flasks and remaining cells in accordance with local safety regulations.
15. Pellet the cell debris by centrifugation at 500 *g* for 10 min at 4°C.
16. Transfer 200–1000 μL aliquots of the supernatant into screw cap tubes.
17. Transfer a 500 μL aliquot of the mock control supernatant into a screw cap tube.
  a. The rest of the mock control supernatant can be discarded.
18. Store the aliquots at −80°C.

***Note:*** The infectivity of the stock decreases with freeze thawing. For maximum accuracy, store the aliquots overnight (16 h) or longer at −80°C before continuing with the next step. Adjust the volume of the aliquots to avoid subsequent freeze thaw cycles. 200 and 500 μl aliquots are good starting points.

### Determining the infectious particles by plaque assay

**Timing: [4 days]**

This step is used to quantify infectious SARS-CoV-2 particles per mL of virus stock. A 10-fold serial dilution of the original stock is added to confluent cell monolayers and overlaid with a viscous medium to restrict diffusion of progeny virions. This prevents convective spread of the virus, so each infectious particle generates a single plaque. Counting of the plaques at different dilutions can be used to estimate the number of infectious particles in the original sample.

19. Plate 5**×**10^5^ Vero E6 cells in 2 mL complete growth medium per well of a six-well plate. Incubate overnight (16 h) at 37°C with 5% CO_2_. Prepare 1 plate per virus stock to be quantified and one plate for the mock control supernatant.
  a. Alternatively, plate 1.5**×**10^5^ Vero E6 cells in 2 mL growth medium per well of a six-well plate. Incubate 3 d at 37°C with 5% CO_2_. This allows setting up the plates on Friday and continuing with the inoculation step on Monday.
20. Confirm that the cells have formed a confluent monolayer by observing them under the microscope.
21. Virus dilutions can be made in 1.5 mL reaction tubes or alternatively, in 96-well deep block plates (2.2 mls).
22. For 1.5 mL reaction tubes:
  a. Take the 6 1.5 mL reaction tubes for the dilution series required and label with dilution (this may be between 10^−1^ and 10^−6^ for each isolate) and isolate name.
  b. Add 900 μL D2 to each tube/well. Prepare six tubes per virus stock to be titrated.
  c. Transfer 100 μL virus to the first tube/well. Discard tip.
  d. Mix the first virus dilution by pipetting up and down several times.
  e. Transfer 100 μL of this diluted virus into the second tube/well. Discard tip.
  f. Continue tenfold dilution series to 10^6^ dilution.
23. Repeat step 22 for every virus stock and for the mock control supernatant.
24. Aspirate the medium from each well of one 6-well plate.
25. Quickly transfer 0.5 mL of each dilution of each virus into a well. Start with the highest dilution (10^−6^) of virus working up to the lowest dilution. It is not necessary to change tips between dilutions.
26. Incubate at room temperature for 40 min, gently tilting the plates every 5 min to prevent the cell layer from drying out.
27. Add 2 mL overlay medium to each well. There is no need to remove the supernatant.
  a. Mix overlay and inoculum by gently swirling the plate.
  b. Incubate undisturbed at 37°C with 5% CO_2_ for 3 days. Troubleshooting 1
28. Visible plaques are usually observed after 3 days but this can be reduced to 2 days or increased to 4 days depending on the virus strain.
29. Remove the supernatant by aspiration and dispose as contaminated waste.
30. Add 5 mL fixative per well and incubate for a minimum of 1 h at room temperature inside the microbiological safety cabinet.

***Note:*** Adjust this step to conform with local rules for inactivation of the virus and decontamination of the plate.

31. Remove the fixative and add 2 mL of PBS per well.
32. Remove the PBS and add a minimum of 1 mL staining solution to each well. Incubate for ∼4 h or until sufficient staining of the remaining cell monolayer is observed.
33. Remove the staining solution.
34. Gently wash away the remaining staining solution with water.
35. Take care not to apply water with force as this can damage the cell monolayer.
36. Invert the plates and allow the cell monolayer to airdry.

***Optional:*** Image the plate wells.

37. Count wells that contain approximately 10–50 plaques. Check that the 10-fold dilutions agree to approximately 2-fold.
38. Calculate the plaque forming units (PFU) per mL of virus stock as follows: Multiply the obtained number of plaques by the dilution and correct for volume of virus added (e.g., if you obtained 43 plaques in the well to which you added 500 μL of the 10^−5^ dilution, the titer of the original stock is 43 **×** 10^5^ **×** 2 = 8.6 **×** 10^6^ PFU ml^−1^). Troubleshooting 2

### Isolation of human hematopoietic stem and progenitor cells from bone marrow, umbilical cord or peripheral blood

**Timing: approximately 8 h**

39. Make sure to have a separate, new disposal bag for anything that comes into contact with the blood so it can be discarded immediately after finishing.
40. Turn on the hood and spray down with 70% ethanol, along with pipet-aides, tissue culture flasks (T175s), pipettes, etc.
41. Estimate the total volume of fresh blood or bone marrow (BM) aspirate that you have. When using human bone marrow purchased from StemCell Technologies (Cat#70001), you will begin the protocol at this point by defrosting the sample in pure FBS and centrifuge at 300 *g* for 5 min at 20°C and continue with step 52.
42. You will need to dilute the blood or BM 2:1 with PBS (e.g., if you have 150 mL of blood in total, you will need to add 300 mL of PBS).

***Note:*** Diluting the blood 3**×** (e.g., 150 ml of blood with 450 ml of PBS) can give a cleaner layer of mononuclear cells after centrifugation.

43. Pour the blood or BM into a large tissue culture flask and mix with an appropriate volume of PBS.
44. Prepare 15 mL of Ficoll into 50 mL falcon tubes (prepare enough tubes considering you are going to add 30 mL of PBS-diluted blood in each tube).
45. Making sure the blood and PBS are mixed, layer 30 mL of blood very gently so as to not mix it with the Ficoll.

**Figure 2:**
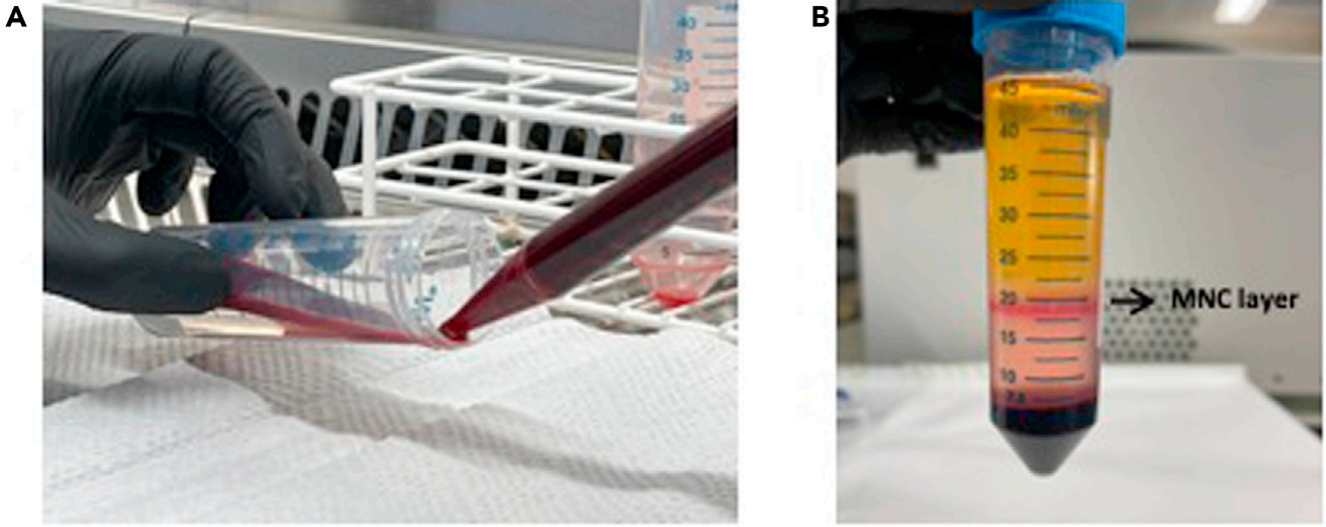
Mononuclear cell isolation using Ficoll gradient Try to pipette 30 mL of blood very gently and angle the tube as horizontal as possible to not mix it with the Ficoll (A). After the centrifugation you should see 4 layers (B).

***Note:*** If there is an option, select the lowest flow setting on the pipette and angle the tube as horizontal as possible without spilling the Ficoll and blood within. Both of these will minimise the risk of the blood mixing in with the Ficoll (Figure 2A).

46. Spin down at 450 *g* for 30 min and at 20°C–25°C, making sure there is no brake on.

**CRITICAL:** Having any type of brake on the centrifuge will disturb the layer of mononuclear cells.

**CRITICAL:** It is imperative that the centrifuge be at room temperature (20°C–25°C) for the Ficoll to retain its correct desity.

***Note:*** After the spin, there should be 4 distinct layers: a bottom layer of dark red blood cells with the Ficoll above, a top layer of serum, and a thin layer in the middle (between the serum and Ficoll) of mononuclear cells. You want to collect the mononuclear cells (Figure 2B).

47. Take the mononuclear cell layer using a pipette and transfer it to a new 50 mL falcon tube. For this, you can pool the mononuclear cell layers from several falcons from the first spin until you have 20–25 mL in total in the new falcon tubes.
48. Dilute 1:1 with PBS+2% FBS (i.e., add 20–25 mL of PBS + 2% FBS to 20–25 mL of mononuclear cell solution) and centrifuge at 300 *g* for 5 min at 20°C.
49. Remove the supernatant from all of the falcon tubes.
50. To lyse the red blood cells resuspend one cell pellet with 20 mL ammonium chloride. Transfer this resuspended cell pellet to the next falcon and use it to resuspend the next pellet. Repeat the process in order to resuspend up to 5 cell pellets in 20 mL ammonium chloride. Leave at 20°C–25°C for 5 min. Troubleshooting 3
51. Add 2 mL of pure FBS (heat inactivated) to buffer the lysis and centrifuge at 300 *g* for 5 min at 20°C.

***Note:*** When using human bone marrow purchased from StemCell Technologies (Cat#70001), you will begin the protocol at this point by defrosting the sample in pure FBS, then centrifuge at 300 *g* for 5 min at 20°C and continue with step 52.

52. Remove the supernatant and resuspend the cell pellet in 5 mL 2% FBS/PBS.
53. Count the cells and continue with the HSPC purification.

**Pause point:** At this point, you can freeze the cells in FBS with 10% DMSO (Mitrus et al., 2018) and continue another day. Just be aware that a cycle of freeze/thaw will reduce your yield of isolated cells considerably. Troubleshooting 4

54. Use the EasySep Human Progenitor Cell Enrichment kit (StemCell Technologies, Cat#19356)
  a. Prepare your mononuclear cell suspension at 5 **×** 10^7^ cells per mL in 5 mL polystyrene tubes in a maximum volume of 2 mL.
  b. Add 50 ul/mL of Enrichment Cocktail to the sample and incubate at 20°C–25°C for 15 min.
  c. Add 100 ul of Magnetic particles per mL of sample and incubate at 20°C–25°C for 5 min.
  d. Top up to 2.5 mL with 2% FBS/PBS and place the tube into the magnet and incubate at 20°C–25°C for 10 min. The magnet draws out the magnetic beads which are attached to the lineage cells to be discarded.
  e. Carefully transfer the non-magnetic cell suspension with the pipette into a new 5 mL polystyrene tube and centrifuge at 300 *g* for 5 min at 20°C.

55. Stain the enriched cell suspension with the antibody cocktail listed in key resources table to define and sort for hematopoietic stem cells and erythroid progenitors. This should be carried out using the following steps:
  a. Prepare the antibody cocktail in the following way (antibody dilutions together with the identifier codes can also be found in the key resources table), mixing the cocktail thoroughly via pipetting:
  b. As indicated above, each sample should be stained in a solution made up of the flow buffer (2% FBS in PBS) and the antibodies.
    We recommend making a master mix for all of your samples. In order to do this, multiply the volumes of each reagent in the table above by N+1, where N is the number of samples you have to ensure you have enough staining volume for the last sample. E.g. if you have 10 samples, multiply the staining volume by 11 to produce the master mix for the antibody cocktail.
    ***Note:*** We recommend titrating the staining volumes of each antibody prior the execuation of the experiment as the optimal titers may vary according to the number of cells that are to be stained and the level of expression of each marker/antigen within those cells. As with the master mix, the reagent volumes should be multiplied up according to the total volume used per sample (e.g. multiply all of the reagent volumes in the table above by a factor of 3 if it is determined that 300 uL of staining volume is needed per sample).
    **CRITICAL:** You must ensure that the antibody vials are opened and handled without direct exposure to light (e.g. switch off the light of the fume hood you are working in), to avoid photobleaching of the conjugated antibodies.
  c. Centrifuge the polystyrene tubes with the non-magnetic cell suspension (from step 54e) for 5 min at 300 *g* and 20°C.
  d. Remove the supernatant from the sample tubes and add the necessary volume of the antibody cocktail per sample.
  e. Resuspend the pellet of cells in each tube in the antibody cocktail via pipetting.
  f. Incubate at 4°C for 30–60 min, covering with aluminum foil if necessary to protect from light.
  g. After the incubation period finishes, wash the samples by adding the flow buffer (see Table 1 for details) at a volume which is at least 10-fold the staining volume e.g., at least 1 mL flow buffer for 100 uL staining volume.

**Table 1:** Reagents and relative volumes to use in order to stain samples for fluorescence assisted cell sorting

Reagent	Volume (per 10^6^ cells) in μL
Lineage cocktail-APCCy7	1 (of each Ab)
CD34-PECy7	1
CD38-PE	2
CD117-BB700	1
CD71-APC	1
CD235a-FITC	1
Flow buffer: 2% FBS in PBS	94
TOTAL	100
  h. Centrifuge the samples for 5 min at 300 *g* and 20°C.
  i. Prepare the solution to be used for staining of the viability marker. For this, dilute DAPI (BD Biosciences, Cat#56407) 1:1000 in the flow buffer.
    ***Note:*** As with the staining volume, the volume of the DAPI solution to be used per sample should be titrated according to the number of cells present and to ensure an appropriate concentration of cells in the solution for the purposes of an efficient sorting process.
  j. Remove the supernatant from the sample tubes and resuspend the pellets in the DAPI solution via pipetting.
  k. Filter the samples in order to remove any clumps in the cell suspension that may block the sorter.

**Figure 3:**
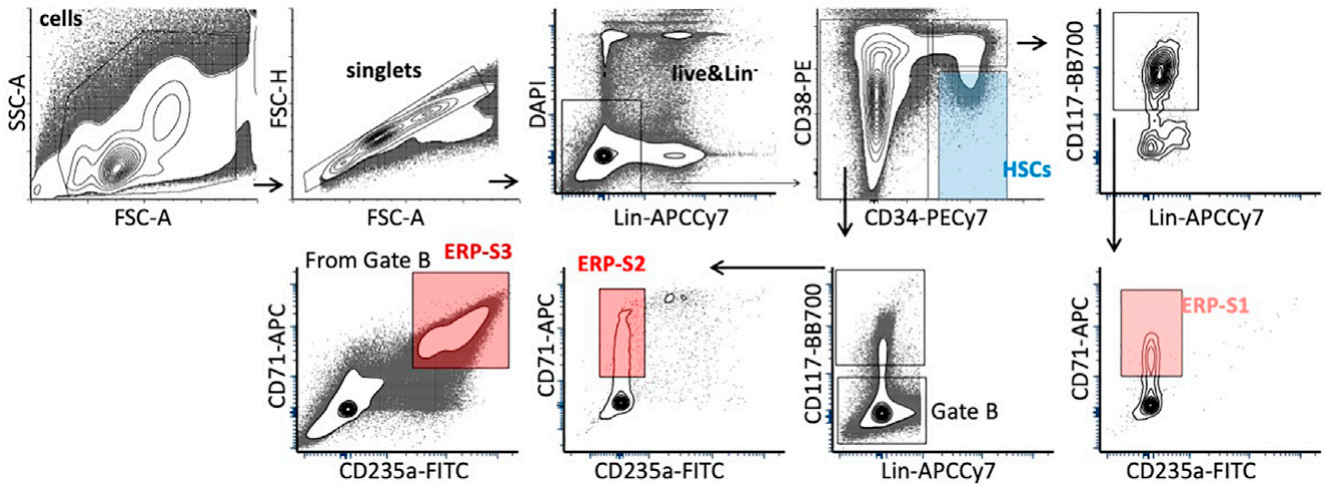
Representative gating strategy to define hematopoietic stem cells and erythroid progenitors

***Note:*** PBS+2% FBS can be used as collection media but you achieve better recovery of cells with pure FBS.

56. Prepare 4 × 1.5 mL Eppendorf tubes for Fluorescence-Activated Cell Sorting (FACS) collection of each sample by adding 200 μL of pure FBS into each tube. Up to 4 populations can be sorted simultaneously, as available instrumentation allows (e.g., HSCs, ERP-S1, ERP-S2 and ERP-S3 as in Figure 3).

57. Centrifuge the sorted cell populations at 300 *g* for 5 min at 20°C and resuspend in StemSpan SFEM (Stem Cell Technologies) supplemented with 100 ng/mL rhFLT-3L, 100 ng/mL rhSCF and 100 ng/mL rhTPO (e.g., 5,000 cells resuspended in 400 μL in a 48-well plate). Troubleshooting 5

### Infection of hematopoietic stem and progenitor cells (HSPCs) with SARS-CoV-2

**Timing: 24–30 h**

58. Infect Hematopoietic cells with SARS-CoV-2 (add the virus directly into the well) at a MOI of 0.5 or 5 PFU per cell for 30 min (to analyze binding capacity of the virus to cells) or 24 h (to analyze active viral infection) in the incubator (37°C, 5% CO_2_).
59. Transfer cells from the 48-well plate to 1.5 mL Eppendorf tubes and wash three times with PBS (4 min at 300 *g*) to remove unbound virus prior to lysis for RNA isolation (step 60) or to culture in methylcellulose for a colony forming unit (CFU) assay (see step 64).

### SARS-Cov-2 quantification by reverse transcription quantitative real-time PCR (RT-qPCR)

**Timing: 6–8 h**

60. After removing the last PBS wash, lyse the pellet with RLT buffer from the RNeasy Microkit (Qiagen, Cat# 74004) and vortex thoroughly.

**Pause point:** Samples can be stored in RLT buffer at −15°C to −25°C for 3–4 weeks.

61. Perform RNA isolation according to the manufacturer’s instructions.

***Note:*** To minimize loss of material, elute by adding 13 μl elution buffer. As the dead volume of the column is 2 μl, this will result in 11 μl of eluted RNA, which is the maximum volume recommended in the reverse transcription reaction.

62. Use all 11 μL of RNA to perform cDNA synthesis using SuperScript III First-Strand Synthesis system (Thermo Fisher Scientific, Cat# 18080051).

***Alternatives:*** Transcription First Strand cDNA synthesis kit (Roche, Cat#04896866001).

**Figure 4:**
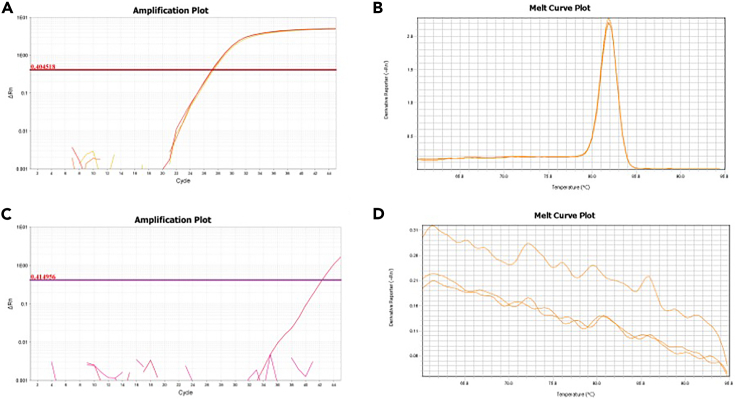
SARS-CoV-2 quantification by RT-qPCR Representative Amplification plots (A and C) and Melting Curve plots (B and D) from viral gene N. Samples that are positively infected show Ct values of 20–28 in the amplification plot (A) and clear and uniform peak in the melting curve plot (B). Notice that samples that are not infected could give a late signal, above 35 Ct (C) due to background noise (D).

***Note:*** We recommend using 384-well reaction plates and performing the reaction in 10 μl volume to maximize the number of reactions and target genes you can run.

63. Analyze SARS-CoV-2 detection by quantitative real-time PCR using the primers described in the key resources table (Figure 4).

### Colony forming unit assay and viral quantification in colonies

**Timing: 10–14 days**

64. (From step 58). After 24 h of infection, 5,000 cells will be seeded for 10–14 days in a methylcellulose-based medium with recombinant cytokines for human cells (StemCell Technologies, Cat#04435) following these steps:
  a. Incubate the different hematopoietic stem and progenitor cells during the 24 h infection period in 48 well-plates, using 400 μL media (same conditions as in step 58).
  b. After 24 h of infection, add 1 mL of methylcellulose to the media using blunt end needles.
  c. Thoroughly mix the cells in the media with the methylcellulose using a 2 mL syringe.
  d. Take all the methylcellulose mix with the syringe and divide into two wells of 12 well-plates, adding 700 μL into each well.

***Note:*** Add 2 mL PBS into three empty wells of the 12-well plate, to prevent the methylcellulose from drying out during the incubation period. If this occurs, then it will make picking the colonies very difficult.

65. Incubate for 10–14 days in the incubator (37°C, 5% CO_2_).

**Figure 5:**
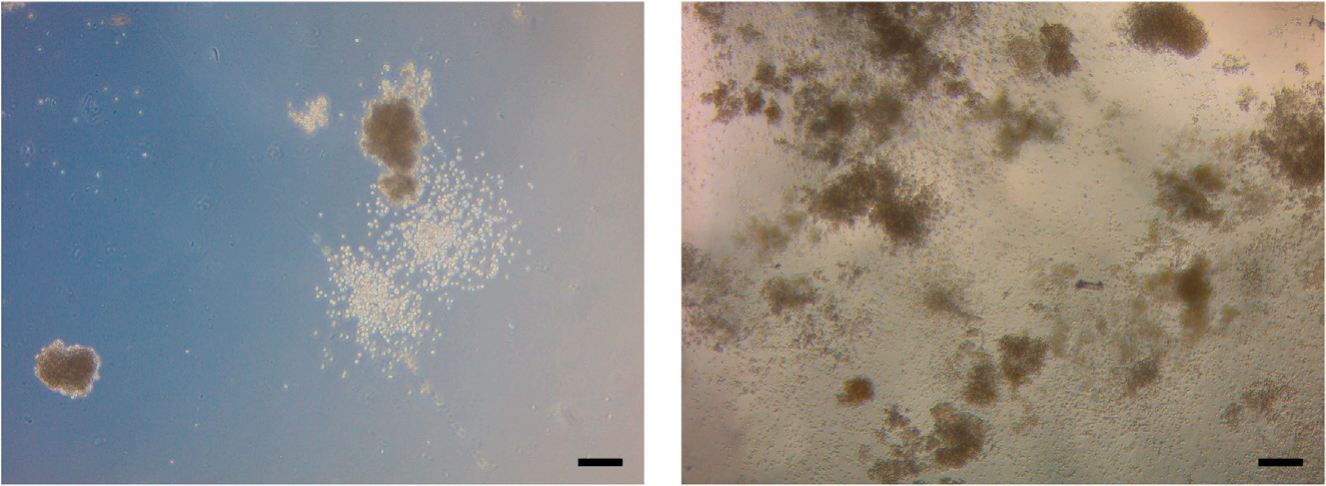
Colony forming unit assay after SARS-CoV-2 infection Representative examples of appropriate colony density to pick individual colonies (left) and overconfluent colony plate (right) where it is difficult to distinguish and pick indiviudal colonies. Scale bar 100 μm.

**CRITICAL:** Colony density must be appropriate for picking individual colonies. Start screening the colony plates under the microscope by day 7 to ensure that you can pick individual colonies (Figure 5). Troubleshooting 6

66. Check the colonies under the microscope to count, score and pick them. For details about the type of colonies to expect, please refer to (Huerga Encabo et al., 2021).
  a. Using the microscope make sure you choose the appropriate slide holder e.g., the adjustable version for the individual plates and the larger rectangular version for the multi-well plates used for counting.  
    ***Note:*** If individual colonies are spread and visible without using a microscope you can directly pick the colonies.
  b. Mark the plate with a pen at the bottom (i.e., at 6 o’clock) as this will be useful when rotating the plate at the end to check for colonies on the borders- you will know when you have finished as the marks will be in the same position as at the start.
  c. Start counting from the top left of the plate and work across. Then when reaching the other side, go down (making sure there is a little overlap from the 'row' above) and work across from right to left. Repeat this process to cover the entire area of the plate. 
  d. Acording to the number of colonies counted, prepare 1.5 mL Eppendorf tubes with 350 μL of RLT buffer from the RNeasy Microkit (Qiagen, Cat# 74004) in each. One tube will be used for every individual colony picked, so that they can be placed directly into the RLT buffer.
  e. Pick and score each colony, using a p10 pipette set at 10 μL.  
  f. Transfer the colony/methylcellulose mixture into the RLT buffer by pipetting up and down repeatedly. Then press all the way down to fully dispense from the pipette tip, before discarding the tip and changing to the next one.  
  g. Work your way around the plate doing this until you reach the bottom. 

***Note:*** Once done, you need to check the borders: start in the top left corner again and then rotate the plate, focusing on the same part of the plate the whole time. The idea is to find more colonies on the borders which may have been previously obscured by the margins of the plate holder. Using the mark that was made earlier, you will know once you have fully rotated the plate.

67. Proceed to the RNA extraction, retrotranscription and RT-qPCR (as done in steps 61 and 62) for the viral quantification from the different colonies

## Expected outcomes

Using SARS-CoV-2 England/02 we routinely obtain stocks with titers in the high 10^6^ to low 10^7^ PFU ml^−1^ range. This might differ depending on the variant.

We tend to obtain 100–150 **×** 10^6^ mononuclear cells per 50 mL of freshly acquired peripheral or cord blood (processed within 24 h of sampling the cord blood from the donor).

## Limitations

There may be variability in the exact yield of mononuclear cells and therefore haematopoietic stem and progenitor cells extracted from the blood or bone marrow source selected. This will in part be influenced by the quality of the source (especially how long after its acquisition it is processed or the thawing of frozen samples) and the efficiency of FACS.

## Troubleshooting

### Problem 1

Irregular shaped plaques in the plaque assay (step 27 of step-by-step method details).

### Potential solution

This generally indicates that the plates have been moved during the incubation time. If the plaques appear to have comets this is usually caused by small movements during the incubation period. Check that the incubator is placed on a solid surface and not exposed to vibrations. Do not slam the doors of the incubator shut.

### Problem 2

Low titer of virus stocks (step 38 of step-by-step method details).

### Potential solution

(1) Ensure that you harvest the virus containing supernatant at the optimal time as explained in step 12. Prepare several infected flasks and harvest at different time points to obtain the best titer. (2) Different virus variants might have different requirements for expression levels of hACE2 and hTMPRSS2. Obtaining Vero cells that constitutively overexpress one or both of these proteins can improve stock titers of some variants. (3) Accumulation of defective interfering particles during consecutive passages. Prepare an early passage inoculation stock and use this stock for the preparation of your working stock. Make sure that you infect cells at on MOI of 0.01 PFU per cell or lower.

### Problem 3

Incomplete lysis of red blood cells during the protocol to obtain HSPCs (step 50 of step-by-step method details).

### Potential solution

There are four steps to take: (1) Try increasing the ratio of ammonium chloride to cells being lysed, for example by resuspending fewer cell pellets (three pellets instead of five) per each 20 mL of ammonium chloride. (2) Leave the pellets to resuspend for a longer time period in the ammonium chloride, for example ten instead of five minutes. (3) If the previous two measures do not work, change the batch of ammonium chloride if possible as the issue may be batch specific. (4) Alternatively, you can also incubate the cells with the lysis buffer in a water bath at 37°C.

### Problem 4

Poor yield of mononuclear cells from blood or bone marrow source (step 53 of step-by-step method details).

### Potential solution

A poor yield would be defined as being below the expected 100–150 **×** 10^6^ mononuclear cells per 50 mL blood. As mentioned under ‘limitations’, it is important to process a source that is recently acquired (within the past 24 h) and avoid freeze-thaw the sample.

Within the protocol, there are a couple of solutions: (1) Further dilute the blood with PBS (step 4 of step-by-step method details) beyond the 2:1 ratio, for example trying a 3:1 or 4:1 ratio to increase separation during the Ficoll spin; (2) Ensure that all of the layer containing mononuclear cells is removed (step 9 of step-by-step method details), returning several times with the pipette to do so if necessary.

### Problem 5

Poor yield of HSPCs (step 57 of step-by-step method details).

### Potential solution

Ensure that an accurate count of the mononuclear cells has been performed prior to using the Human Progenitor Cell Enrichment Kit. This will mean that a sufficient volume of reagents (e.g., the selection cocktail and magnetic particles) from the kit are used for the number of cells you have. Also be sure to filter the mononuclear cells before carrying out the enrichment as they may form clumps which can interfere with the magnetic selection process.

### Problem 6

Colonies are either too sparse or too densely populated to recover a significant yield (step 65 of step-by-step method details).

### Potential solution

In either case, refer back to the number of cells seeded per well as this may need to be adjusted accordingly if the colony density becomes a consistent issue.

Consider seeding a range of cells either below or above the recommended 5,000 cell number and incubating for 10–14 days, to determine which dose is most appropriate for you.

If there are concerns about colonies being too densely populated, you can also monitor the wells of methylcellulose from day 7 onwards and decide to pick them earlier within the 10–14 days incubation period if they reach a suitable level of density.

## Resource availability

### Lead contact

Further information and requests for resources and reagents should be directed to and will be fulfilled by the lead contact, Dominique Bonnet (dominique.bonnet@crick.ac.uk).

### Materials availability

SARS-CoV-2 can be supplied following MTA agreement.

## Data Availability

This study did not generate datasets or codes
